# Assessing the prognostic value of serum creatinine to cystatin C ratio in stage III-IV colorectal cancer: development of a nutritional prognostic scoring system

**DOI:** 10.3389/fonc.2025.1667472

**Published:** 2025-10-21

**Authors:** Weicheng Ji, Jing Zhang, Jiawei Song, Yu Jiang, Yajie Guo, Shuai Liu, Yihuan Qiao, Yanjiang Pei

**Affiliations:** ^1^ General Surgery Department, Xidian Group Hospital, Xi’an, China; ^2^ Department of Endocrinology, No 908th Hospital of Chinese PLA Joint Logistic Support Force, Nanchang, China; ^3^ Department of Gastrointestinal Surgery, The Chengdu Medical College Second Affiliated Hospital, Nuclear Industry 416 Hospital of Nuclear Industry, Chengdu, China; ^4^ Department of Digestive Surgery, Xijing Hospital of Digestive Diseases, Fourth Military Medical University, Xi’an, China; ^5^ State Key Laboratory of Holistic Integrative Management of Gastrointestinal Cancers and National clinical Research Center for Digestive Diseases, Xijing Hospital of Digestive Diseases, Fourth Military Medical University, Xi’an, China; ^6^ Department of Digestive Surgery, Honghui Hospital, Xi’an Jiaotong University, Xi’an, China

**Keywords:** nutrition, serum creatinine, cystatin C, advanced colorectal cancer, prognosis, restricted cubic spline

## Abstract

**Background and aim:**

For patients with stage III-IV colorectal cancer (CRC), malnutrition and poor prognosis are prevalent; however, the prognostic value of the serum creatinine to cystatin C ratio (CCR) in this setting remains uncertain. This study aims to investigate the prognostic significance of CCR and develop a nutritional prognostic scoring system based on CCR.

**Methods:**

Restricted cubic splines (RCS) were utilized to investigate the relationship between CCR and prognosis. Patients were categorized into high and low CCR groups based on RCS cut-off values and divided into quartiles (Q1 to Q4). Kaplan-Meier (KM) curves were employed for survival analysis, and multivariate Cox regression analyses were identified prognostic factors and to construct a tumor nutritional prognostic scoring system for patients. Receiver Operating Characteristic (ROC) and area under curve (AUC) were used to evaluate the scoring system.

**Results:**

The RCS analysis showed a linear relationship between CCR and prognosis (P = 0.76), with lower CCR levels correlating with higher hazard ratios (HR). Clinically, patients in the low CCR group exhibited poorer overall survival (OS), more advanced stages, and a higher proportion of deficient Mismatch Repair status. KM curves revealed that patients with higher CCR levels had better prognoses, with the best outcomes in the Q4 group and the worst in the Q1 group. After adjusting for confounding biases, TNM stage IV (HR = 2.34, 95% CI: 2.03 - 2.70), low CCR (HR = 1.16, 95% CI: 1.01 - 1.33) and CEA (HR = 1.17, 95% CI: 1.13 - 1.22) were identified as risk factors for prognosis, while Body Mass Index (BMI; HR = 0.98, 95% CI: 0.96 - 1.00) and albumin (HR = 0.99, 95% CI: 0.97 - 1.00) were protective factors. Finally, based on albumin levels, BMI, CEA, and CCR levels, a personalized nutritional prognostic scoring system was developed, to predict 1, 2, and 3-year OS, demonstrating good accuracy (AUC = 0.73) and calibration.

**Conclusion:**

CCR levels are closely related to the prognosis of stage III-IV CRC patients, with low levels linked to malnutrition and worse outcomes. We developed a novel nutritional prognostic scoring system, which holds clinical value in predicting outcomes for patients with metastatic CRC.

## 1 Introduction

Colorectal cancer (CRC) is a serious threat to public health, with the third common incidence and second mortality rates among all cancers (1). Nowadays, with the advancement of concepts, the treatment of CRC has become a multifaceted and multidimensional comprehensive treatment mainly based on surgery, supplemented by radiotherapy, chemotherapy, immunotherapy (2–5). However, therapeutic effects are still unsatisfactory for advanced and metastatic (TNM stage III- IV) patients (6). Therefore, it is important to accurately predict clinical prognoses of patients with advanced stage and intervene timely for them.

Advanced cancer patients, many studies have been conducted on their gene mutations, tumor microenvironmental conditions, protein expression alterations and changes in intestinal gut (7–10). In addition, as a tumor of the digestive system, CRC considerably impacts the nutritional status of patients and further affects their life quality (11). Therefore, we should accurately and timely evaluate their nutrition after the cancer diagnosis. Among the assessment of nutrition, skeletal muscle loss is essential, however, which requires specific imaging instruments to determine, such as ultrasound (12), Magnetic Resonance Imaging (MRI), Computed Tomography (CT), bioelectrical impedance analysis, and dual-energy X-ray absorptiometry (13); Tests of muscle strength and somatic function can be time-consuming and cumbersome to perform. Additionally, their inability to support continuous monitoring can pose challenges in achieving widespread popularity.

Nutritional and muscle status assessment in CRC patients, particularly those with advanced disease, is challenging in clinical practice. Traditional methods such as the Prognostic Nutritional Index (PNI) and the Controlling Nutritional Status (CONUT) are influenced by factors like inflammation, making them less reliable for continuous monitoring (14). Imaging techniques like CT and MRI can measure muscle mass but are expensive and not routinely available. Given these challenges, there is a need for a simpler, more accessible biomarker. Cystatin C (CysC) is exclusively eliminated through glomerular filtration and remains largely uninfluenced by dietary habits, body composition, or individual variability (15). Similarly, while albumin is widely used as an indicator of nutritional status, it is also affected by systemic inflammation, making it less reliable in the context of cancer. Recent studies have demonstrated that in patients with normal renal function, the primary factor differentiating serum creatinine and cystatin C levels is the patient’s skeletal muscle mass (16). In other words, the ratio of serum creatinine to cystatin C (CCR) is closely associated with the onset of skeletal muscle loss (17–19). Several studies have validated the relationship between CCR and prognosis for tumor patients (19–21). Skeletal musculoskeletal disorders are prevalent in more than 80% of stage III-IV patients due to factors such as the tumor metastasis and chemotherapy (11, 22). The CCR offers a promising solution, as it can be easily measured through routine blood tests and reflects muscle mass, making it a practical tool for clinical use. Early identification and intervention are crucial for patients with stage III - IV CRC, but few studies have analyzed the relationship between CCR levels and prognosis among advanced CRC.

This retrospective study delved into the association between CCR levels, and the overall survival (OS) rates of individuals diagnosed with stage III-IV CRC, aiming to establish a nuanced, nutrition-focused scoring mechanism for the prognostication of each patient’s outcome.

## 2 Population and methods

### 2.1 Population

A total of 3036 CRC patients who underwent surgical treatment were retrospectively enrolled at the First Affiliated Hospital of Air Force Military Medical University from 2013 to 2019. The Inclusion criteria were as followed: (1) patients with CRC with pathological staging of stage III or resectable stage IV; (2) age 18–80 years; (3) patients with detailed clinical features and pathological examination. The exclusion criteria were: (1) combined with other primary tumors; (2) patients with special types of CRC, including Lynch syndrome and hereditary CRC, due to their distinct genetic and molecular characteristics, which may confound the relationship between CCR and prognosis in sporadic CRC; (3) abnormal renal function and combined with other renal diseases, as these conditions may interfere with the assessment of CCR levels; (4) patients combined with serious complications and died within one month after surgery; (5) Patients with inoperable distant metastases, as their prognosis and treatment differ significantly from those with resectable disease; (6) the number of lymph nodes obtained by surgery is less than 12; (7) the follow-up time is less than one month, to ensure sufficient data for survival analysis; (8) combined with intestinal obstruction or perforation of the tumor site; (9) pregnant women. This trial was approved by the Ethics Committee of the First Affiliated Hospital of the Air Force Military Medical University, approval number KY20232232-F-1, and registered in the China Clinical Trial Registry, registration number ChiCTR2300075253.

### 2.2 Clinical characteristics

Patient baseline information (sex, age, height and weight), laboratory test results (blood routine, liver and kidney function), surgical pathology information (tumor location, surgical method, TNM stage, Mismatch Repair (MMR) status), tumor markers (AFP, CEA, CA125, CA199, CA724), nutritional status (NRS2002 score), and physical fitness scores were collected. The hematological analysis consisted mainly of routine blood tests, liver and kidney functions, etc., which were analyzed using a fully automated instrumental hematology analyzer. Tumor markers, including AFP, CEA, CA125 and CA199 levels, were determined using enzyme-linked immunosorbent assay kits.

Surgery for CRC patients was performed according to the principle of Total Mesorectal Excision (TME) or Complete Mesocolic Excision (CME), with resection of the entire bowel segment plus regional lymph node (LN) dissection, and the surgical types mainly included: laparoscopic and open surgery. MMR status was determined by immunohistochemical detection of MLH1, MSH2, MSH6 and PMS2 expression. The presence of deletion of expression of any of these proteins was classified as deficient MMR (dMMR) and the absence of deletion of expression of all proteins was classified as proficient MMR (pMMR). The formula for calculating CCR was calculated by serum creatinine (mg/dL)/CysC (mg/L) (20).

Patients were followed up every three months in the first two years postoperatively and every six months thereafter. The primary endpoint of this study was OS, which was defined as the time interval between the patient’s diagnosis and the date of death from any cause or the date of the last follow-up visit.

### 2.3 Statistical analysis

Data were analyzed using R4.2.3 software. Measurement was expressed as mean ± standard deviation (X ± SD) if it conformed to normal distribution, and t-test was used for comparison between groups; if it did not conform to normal distribution, it was expressed as median ± with interquartile range (median ± IQR), and non-parametric test was used for comparison between groups. The qualitative data were expressed as rates (%) and comparisons were made using the χ^2^ test. A restrictive cubic spline (RCS) function was applied to present linear or nonlinear prognostic profiles of CCR and its cut-off was determined when HR = 1. For survival analyses, Kaplan-Meier (K-M) curves were plotted to compare survival differences, detected by the log-rank test. The hazard ratios (HR) and 95% confidence intervals (CI) were used to assess prognostic risk factors. In the model construction, we first performed univariate Cox regression to select variables with a P-value < 0.05, and then included these variables in the multivariate Cox regression analysis for modeling. Survival prediction columns were plotted based on the Cox regression model, where the bar values for each variable represent its weight coefficient in the model. The calibration curves and Receiver Operating Characteristic curve (ROC) were plotted to assess their accuracy. P < 0.05 indicates a statistically significant difference.

## 3 Results

### 3.1 Patients

The study enrolled a total of 3036 patients with a mean age of 60 ± 8 years, of whom 1740 (57.3%) were male and 1296 (42.7%) were female; 1632 (53.8%) laparoscopic surgery and 1404 (46.2%) open surgery; 1385 (45.6%) colon cancer and 1651 (54.4%) rectal cancer; 219 (7.2%) patients with dMMR and 2817 (92.8%) patients with pMMR. The TNM stage was shown in the **Table 1**. The survival rates of advanced CRC at 1, 2, and 3 years were 90.8%, 75.3%, and 65.7%, respectively. The median follow-up time was calculated using the reverse Kaplan-Meier method to account for variable follow-up periods among patients. The overall median follow-up was 47.52 months (95% CI: 45.30 – 48.69), with the low CCR group having a median follow-up of 46.32 months (95% CI: 42.33 – 50.68) and the high CCR group having a median follow-up of 48.72 months (95% CI: 46.67 – 50.86). The log-rank test comparing the follow-up periods between the low and high CCR groups yielded a P-value of 0.46, indicating no significant difference in follow-up time between the two groups.

**Table 1:** Basic clinical characteristics.

Characteristics	High	Low	P value
	*N=1553*	*N=1483*	
Tumor site:			0.007
Colon cancer	671 (43.2%)	714 (48.1%)	
Rectal cancer	882 (56.8%)	769 (51.9%)	
gender:			<0.001
female	576 (37.1%)	720 (48.6%)	
male	977 (62.9%)	763 (51.4%)	
age	57.0 [49.0;65.0]	63.0 [54.5;71.0]	<0.001
NRS2002:			0.733
0	156 (10.0%)	159 (10.7%)	
1	609 (39.2%)	572 (38.6%)	
2	412 (26.5%)	411 (27.7%)	
3	376 (24.2%)	341 (23.0%)	
Caprini:			0.778
0-2	287 (18.5%)	289 (19.5%)	
3-4	328 (21.1%)	309 (20.8%)	
>=5	938 (60.4%)	885 (59.7%)	
ECOG:			0.482
0-1	92 (5.92%)	98 (6.61%)	
>=2	1461 (94.1%)	1385 (93.4%)	
Albumin (g/L)	42.9 [38.3;45.9]	41.1 [36.4;44.4]	<0.001
Immunoglobulin (g/L)	26.1 [23.3;29.4]	26.9 [23.8;30.3]	<0.001
AFP (ng/mL)	2.43 [1.73;3.40]	2.56 [1.80;3.58]	0.018
CEA (ng/mL)	1.65 [1.14;2.50]	1.75 [1.23;2.80]	<0.001
CA199 (U/mL)	14.5 [7.68;30.7]	16.2 [8.54;35.9]	0.005
CA724 (U/mL)	2.99 [1.54;7.95]	3.54 [1.58;9.11]	0.044
CA125 (U/mL)	11.4 [8.14;16.7]	12.4 [8.51;19.8]	<0.001
Hemoglobin (g/L)	130 [111,145]	124 [105,139]	<0.001
Platelet (10^9/L)	212 [169,268]	208 [160,266]	0.042
CCR	1.25 [1.14;1.41]	0.86 [0.75;0.97]	0.000
TNM:			<0.001
III	1166 (75.1%)	989 (66.7%)	
IV	387 (24.9%)	494 (33.3%)	
CCR grade:			0.000
Q1	0 (0.00%)	767 (51.7%)	
Q2	43 (2.77%)	716 (48.3%)	
Q3	752 (48.4%)	0 (0.00%)	
Q4	758 (48.8%)	0 (0.00%)	
MMR status:			0.004
dMMR	91 (5.86%)	128 (8.63%)	
pMMR	1462 (94.1%)	1355 (91.4%)	

NRS2002, Nutrition Risk Screening 2002; Caprini, Caprini Risk Assessment Model; ECOG, Eastern Cooperative Oncology Group performance status; AFP, Alpha-Fetoprotein; CEA, Carcinoembryonic Antigen; CA199, Carbohydrate Antigen 19-9; CA724, Carbohydrate Antigen 72-4; CA125, Cancer Antigen 125; CCR, creatinine to cystatin C ratio; dMMR, Deficient Mismatch Repair; pMMR, Proficient Mismatch Repair.

### 3.2 Different clinicopathological features divided by different CCR subgroups

**Figure 1:**
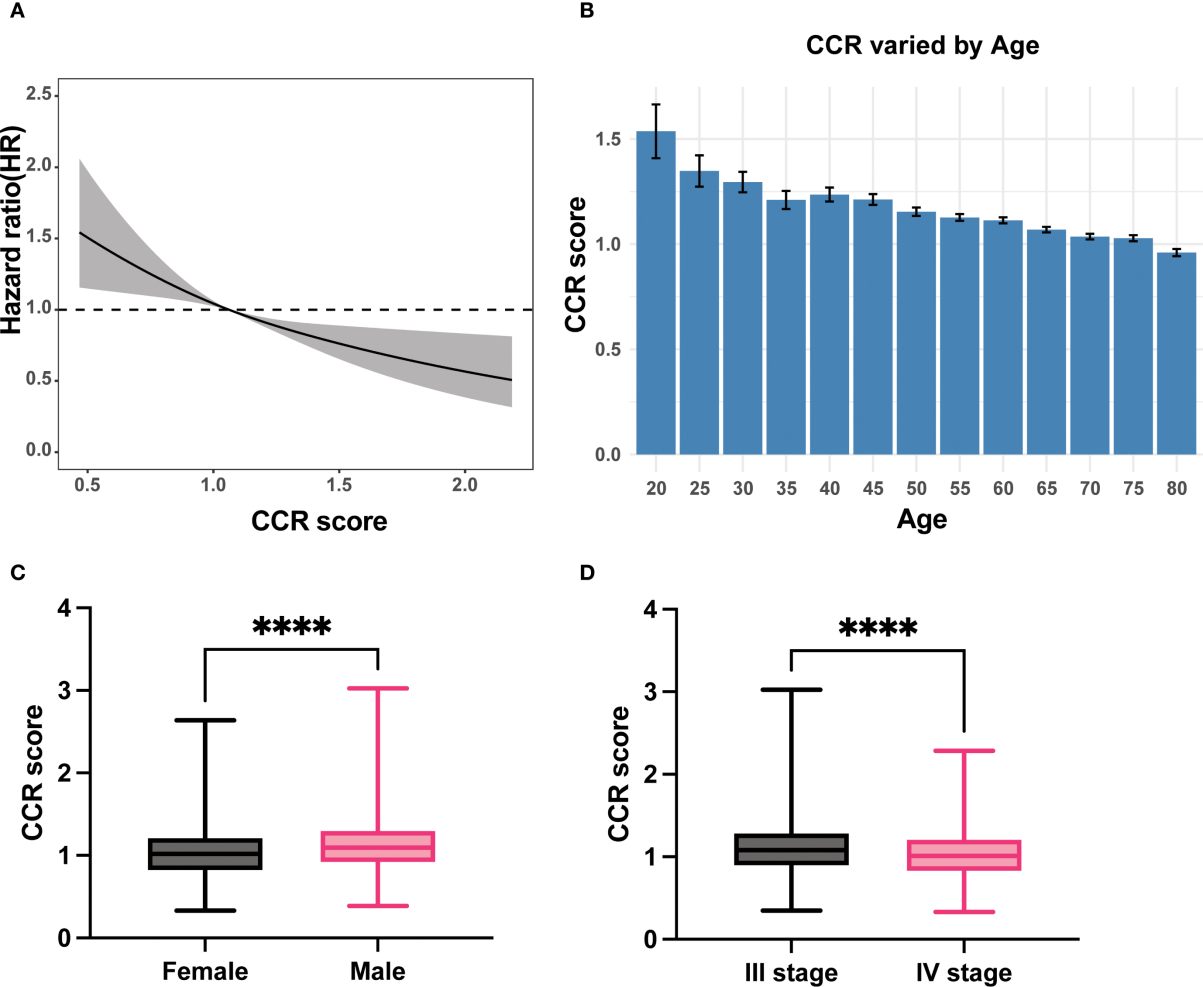
The relationship of CCR with prognosis, age, gender and TNM stage. **(A)** Linear relationship of CCR with OS; **(B)** The levels of CCR varied with the age; **(C)** Male patients had higher CCR levels than female patients; **(D)** Patients with stage III had higher CCR levels than patients with stage IV. CCR: creatinine to cystatin C ratio; OS: overall survival. **** means P < 0.0001.

The patient’s prognosis was found to be directly related to their CCR levels. This relationship was verified using an RCS curve, which showed no significant non-linear correlation (P = 0.76, **Figure 1A**). When the intercept value was set at HR = 1, the cutoff was set as 1.05 and patients was divided into two groups (high CCR and low CCR). The clinical baseline data shows that the high CCR group had a higher proportion of laparoscopic surgery compared to the low CCR group, while the low CCR group had a higher proportion of colon cancer (P < 0.05). The group with high CCR exhibited significantly higher levels of height, weight, Caprini Risk Assessment Model (Caprini), albumin, creatinine, white blood cell (WBC), red blood cell (RBC), hemoglobin (HGB), hematocrit (HCT), platelet (PLT), pLNs and Body Mass Index (BMI; P < 0.05). Conversely, the low CCR group showed an increase in age, immunoglobulin (Ig), CysC, AFP, CEA, CA199, CA724, CA125, N stage, M stage, TNM stage, and dMMR (P < 0.05, **Table 1**). The findings indicate that patients in the high CCR group had better overall nutrition status and earlier stages, but a lower percentage of dMMR.

As individuals age, the glomerular clearance decreases (23), we investigated the correlation between CCR and age. Our results demonstrate that CCR levels decrease as patient age increases (**Figure 1B**).

Subgroups analyses were conducted to validate the clinical characteristics of CCR under different genders due to significant clinical differences in creatinine and renal function between men and women. The results showed that men had significantly higher CCR values than women (**Figure 1C**). Among female patients, the high CCR group showed elevated levels of height, albumin, creatinine, WBC, RBC, HCT, and pLNs, while age, CysC and CEA had decreased levels (P < 0.05). Different surgical procedures were performed on male patients with varying CCR levels (P < 0.05). The CCR-high group exhibited increased levels of weight, albumin, creatinine, WBC, RBC, HGB, HCT, PLT, positive lymph nodes (pLNs), and BMI (P < 0.05), but decreased levels of Ig, CysC, AFP, CEA, CA125, N stage, M stage, and TNM stage (P < 0.05, **Supplementary Table S1**). This study found that stage III patients had higher CCR values than stage IV patients, indicating better nutritional status in stage III patients than stage IV patients (**Figure 1D**).

**Figure 2:**
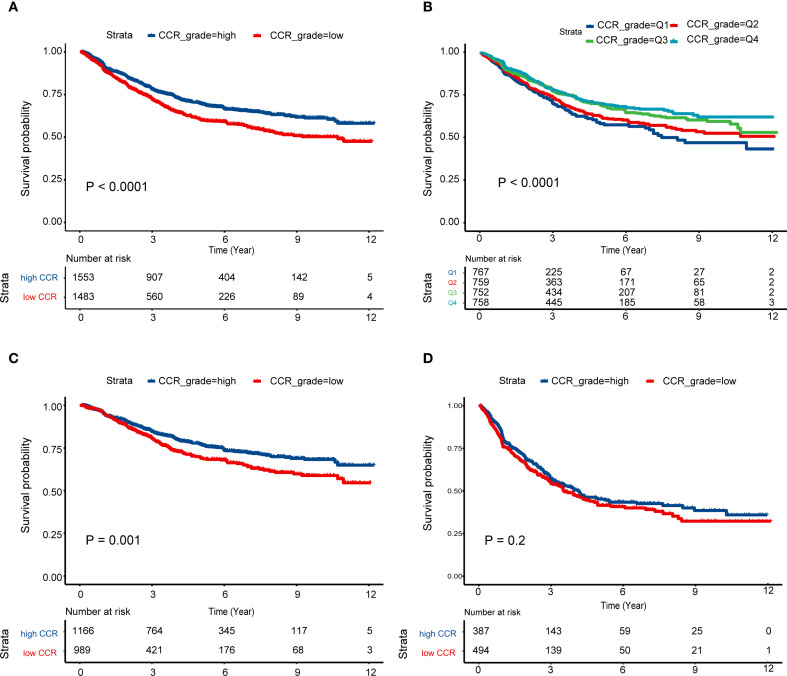
Survival curves of different CCR levels. **(A)** High CCR levels had better prognosis than low CCR levels among the III and IV stage patients; **(B)** Survival differences among the Q1–4 CCR levels; **(C, D)** Survival curves of high and low CCR levels among the patients with stage III **(C)** and IV **(D)**. CCR: creatinine to cystatin C ratio.

Besides, patients were classified into four categories based on quartiles, with Q1 representing the lowest quartile and Q4 representing the highest quartile of CCR. The Kaplan-Meier curves demonstrate that Q4 had the best prognosis (**Figure 2A**), followed by Q3, Q2 had an average prognosis, and Q1 had the worst prognosis (**Figure 2B**). Furthermore, we analyzed the prognosis of stage III subgroups and found that patients with high CCR had a better prognosis (P < 0.05, **Figure 2C**). However, in the stage IV subgroup, there was no significant difference in prognosis between the high and low CCR groups (**Figure 2D**). Among stage III patients, the clinical baseline characteristics showed that patients with different CCR levels had differences in tumor sites. Female patients had lower CCR levels (P < 0.05). The high CCR group had elevated levels of height, weight, albumin, creatinine, WBC, RBC, HCT, and BMI (P < 0.05). Meanwhile, the high CCR group had decreased levels of age, Ig, CysC, AFP, CEA and CA125 (P < 0.05). Among stage IV patients, there were differences in surgical procedures based on varying CCR levels. Female patients exhibited lower CCR levels (P < 0.05). The high CCR group showed elevated levels of weight, albumin, creatinine, RBC, HGB, HCT, pLNs, and BMI (P < 0.05), but age, Ig, CysC, CA724 and CA125 levels were decreased in the high CCR group (P < 0.05, **Supplementary Table S2**).

### 3.3 Cox analysis of advanced CRC patients

The Cox regression model analysis revealed that TNM stage IV (HR = 2.34, 95% CI: 2.03 – 2.70), and CEA (HR = 1.17, 95%CI: 1.13 - 1.22) were risk factors for patient prognosis. Conversely, high CCR (HR = 0.86, 95%CI: 0.75 - 0.99), BMI (HR = 0.98, 95%CI: 0.96 - 1.00) and albumin (HR = 0.99, 95%CI: 0.97 - 1.00) were identified as protective factors for patient prognosis (P < 0.05, **Table 2**).

**Table 2:** Univariate and multivariate Cox analysis for clinical characteristics.

Characteristic	Univariate Cox			Multivariate Cox		
	HR^1^	95% CI^1^	P-value	HR^1^	95% CI^1^	P-value
BMI	0.97	0.95, 0.99	0.005	0.98	0.96, 1.00	0.044
gender						
female	—	—		—	—	
male	0.99	0.87, 1.13	0.893	1.03	0.89, 1.19	0.735
age	1.01	1.00, 1.01	0.001	1.00	1.00, 1.01	0.125
NRS2002						
0	—	—		—	—	
1	0.99	0.78, 1.26	0.942	0.97	0.77, 1.23	0.831
2	1.12	0.88, 1.43	0.361	1.13	0.88, 1.44	0.342
3	1.25	0.98, 1.59	0.078	1.08	0.85, 1.38	0.530
Albumin (g/L)	0.97	0.95, 0.98	<0.001	0.99	0.97, 1.00	0.010
CEA (ng/mL)	1.29	1.25, 1.34	<0.001	1.17	1.13, 1.22	<0.001
HGB (g/L)	0.99	0.99, 1.00	<0.001	1.00	1.00, 1.00	0.857
TNM						
III	—	—		—	—	
IV	2.86	2.50, 3.27	<0.001	2.34	2.03, 2.70	<0.001
CCR grade						
high	—	—		—	—	
low	1.37	1.20, 1.56	<0.001	1.16	1.01, 1.33	0.037

CEA, Carcinoembryonic Antigen; HGB, Hemoglobin; CCR, creatinine to cystatin C ratio; CI, Confidence Interval.

### 3.4 Construction and evaluation of a prognostic nutrition-based nomogram

**Figure 3:**
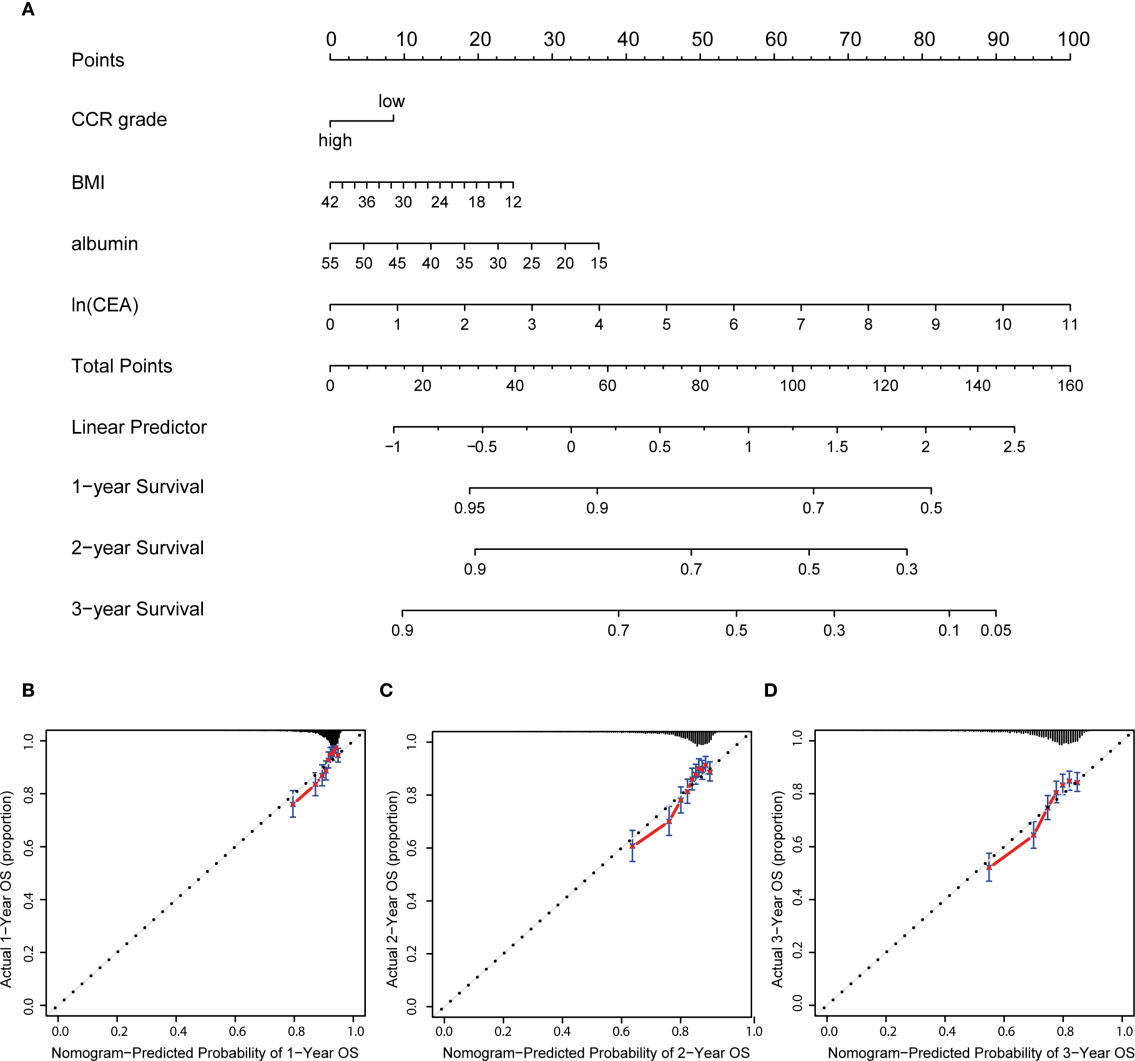
Nutrition-related nomogram and calibration curves. **(A)** Nutrition-related nomogram to predict 1-, 2- and 3-year OS for advanced patients; **(B–D)** calibration cures were plotted to evaluate the predicted probability of 1-, 2-, and 3-year OS; OS: overall survival.

Prognostic nomogram was created, and each patient was assigned a score based on their CCR level, BMI, albumin, and CEA. The nomogram provided 1-, 2-, and 3-year OS based on the total score (**Figure 3A**). The results suggest that the model’s predictions of 1-year, 2-year and 3-year survival are accurate, as the predicted outcomes align well with the actual outcomes (**Figures 3B–D**).

**Figure 4:**
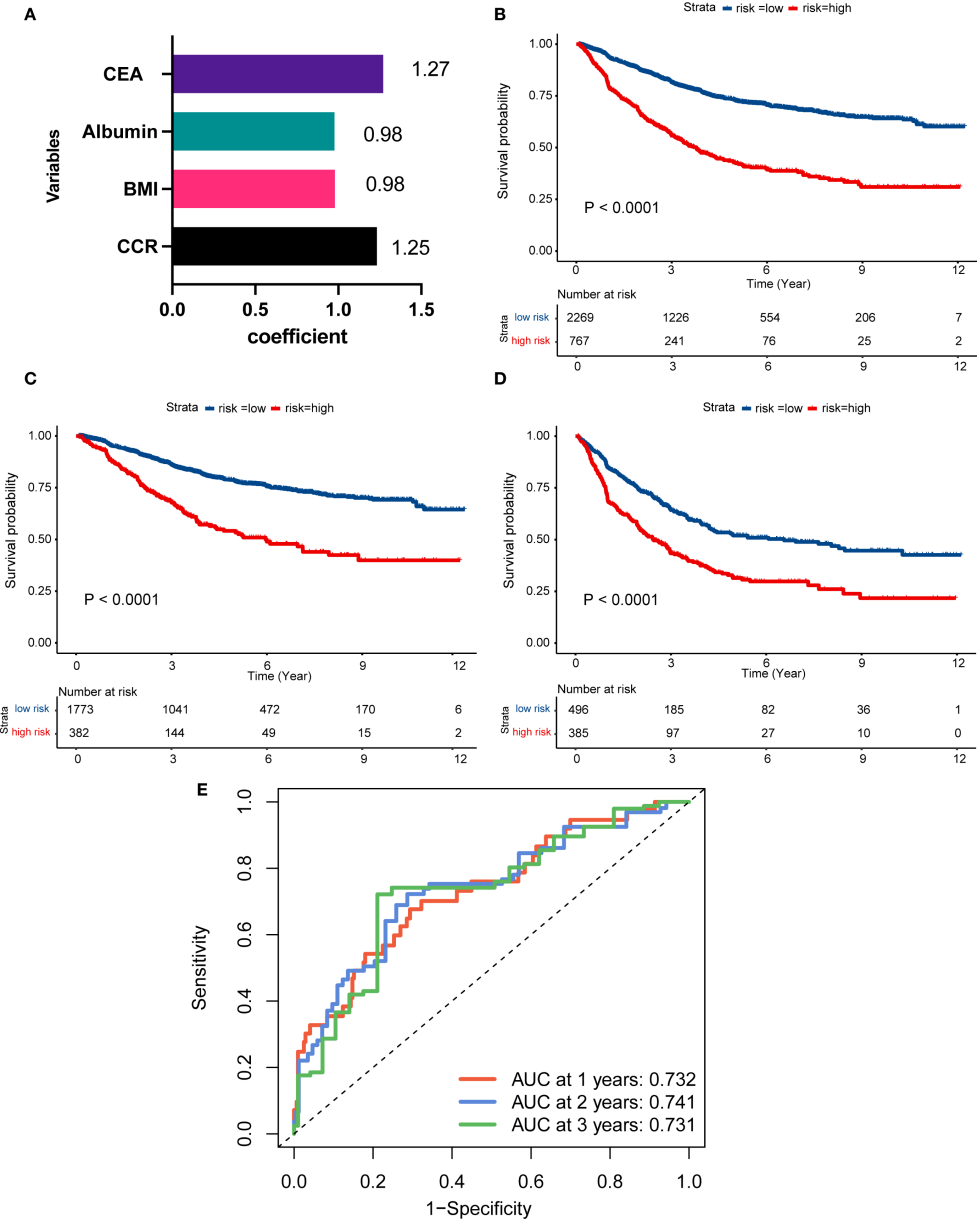
Construction of ABCC simple nutrition system for advanced CRC patients. **(A)** The visualized efficient of CCR, BMI, albumin, and CEA levels of ABCC system; **(B–D)** Patients with high-risk of ABCC system score had worse prognosis than those with low-risk patients among the overall population **(B)**, stage III **(C)** and stage IV**(D)**. **(E)** The ROC curve of the ABCC system in the 1-, 2-, 3-year after surgery. CCR: creatinine to cystatin C ratio; BMI: body mass index; CEA, Carcinoembryonic Antigen; ROC: Receiver Operating Characteristic.

The study introduced an innovative nutritional scoring system, named the albumin, BMI, CEA, and CCR (ABCC) scores. Within the ambit of the ABCC scoring system, BMI and albumin are each attributed a weight of less than 1, reflecting attributes associated with a diminished risk. Conversely, the low CCR level and CEA metric is ascribed a weight greater than 1, signifying its correlation with an elevated risk (**Figure 4A**). Patients were categorized into high- and low-risk groups based on their ABCC scores. By the KM curves, we found high-risk had worse OS than those with low-risk, whether in the advanced stage (**Figure 4B**), in the III stage or IV stage (**Figures 4C, D**), demonstrating the ABCC prognostic system efficiently stratified the risk profiles. The ROC curve for the scoring system demonstrated values of 0.732, 0.741, and 0.731 at 1-, 2-, and 3-years post-surgery, respectively (**Figure 4E**).

## 4 Discussion

Patients with advanced stage (III-IV) have poor nutrition condition and prognosis, due to its tumor characteristics, such as tumor invasion, highly expendable diseases, and tumor metastasis (20). Cachexia, characterized by unintended weight loss and muscle wasting, is a common and detrimental feature in patients with advanced colorectal cancer. Although not directly analyzed in this study, cachexia is often associated with low skeletal muscle mass, which we measured using the creatinine-to-cystatin C ratio (CCR). Emerging evidence suggests that cachexia may serve as a surrogate marker for poor prognosis, as it reflects the systemic effects of cancer progression, including metabolic dysregulation and inflammation. In this study, we verified that CCR was closely associated with clinical outcomes by the RCS curves. Multivariate Cox analysis demonstrated that CCR level, BMI, albumin, and CEA were independent prognostic risk factors for advanced CRC. A prognostic ABCC nutrition-related system score for advanced CRC was constructed based on these four clinicopathological factors with good discrimination.

Nowadays, evaluating patients’ muscle mass required a series of specific examinations that involved large instruments and algorithmic processing. These approaches often fell short of meeting clinical needs (24). Determining the nutrition and muscle mass of patients accurately and efficiently is currently an urgent issue (25). Recently, numerous studies have demonstrated and validated that CCR can be used as an alternative biomarker for skeletal sarcopenia (19, 26). In benign diseases, it has been validated in elderly patients (27), COPD (28) and idiopathic pulmonary fibrosis (29). As for cancers, CCR was strongly associated with the length of ICU stay and patient survival (20, 21). In gastrointestinal tumors, the level of CCR affects the occurrence of postoperative complications and long-term survival in patients with esophageal cancer (21).

Creatine is interconverted with phosphocreatine in skeletal muscle by creatine kinase (30). Phosphocreatine dephosphorylates during exercise to synthesize adenosine triphosphate, which fuels muscle contraction. During rest, creatine regains the phosphate group (31). Creatine and phosphocreatine undergo a non-enzymatic dehydration reaction, leading to their gradual degradation into creatinine. Serum creatinine is mainly produced from phosphocreatine during skeletal muscle metabolism. Therefore, patients with decreased muscle mass have lower creatinine levels. Meanwhile, cystatin C is a small, nonionic protein produced at a constant rate by nucleated cells and is not affected by muscle metabolism (32). CCR correlates better with poor prognosis in cancer patients than serum creatinine, and this correlation has been hypothesized to be due to muscle mass-mediated reasons (20, 33).

This study included 3036 patients with stage III-IV CRC who underwent radical surgical treatment. Advanced gastrointestinal tumors have a greater impact on the nutritional status and skeletal muscle content of the organism, and it was found that patients in the low CCR group had a worse systemic condition, later tumor stage, and poorer prognoses, which was consistent with other studies (20, 21, 33). In another report, among patients with pancreatic cancer, low CCR levels showed poorer prognosis in relapse-free survival and OS (34). For advanced tumor patients underwent Immune checkpoint inhibitors, a recent literature found pre-treatment CCR levels may serve as a predictive biomarker, and high CCR were associated with significantly longer OS (35). For advanced CRC, it is essential to correctly determine the skeletal muscle status (2). The relationship between CCR and prognosis was investigated by RCS curve among patients with advanced CRC. The clinical significance of CCR was also studied according to the dichotomous (low and high CCR levels) and quaternary classification (Q1-Q4 levels).

Secondly, clinical significance and prognostic value of CCR was further verified in male or female subgroups, III or IV stage subgroups. Considering the differences in renal function between genders, we performed subgroup analyses and found the low CCR group in different genders had worse overall systemic conditions, later tumor stages and poor clinical outcomes. For patients with different stages, the prognostic difference between different CCR levels was not significant in the stage IV subgroup. This may be attributed to the advanced systemic nature of Stage IV disease, where tumor burden, chemotherapy effects, and other systemic factors may overshadow the impact of muscle mass on survival (6). Additionally, the treatment heterogeneity in Stage IV patients, with different chemotherapy regimens and targeted therapies, may contribute to the lack of a clear relationship between CCR and prognosis. More importantly, a convenient and accessible ABCC nutritional prognostic score was constructed, which represents the nutritional index of individual patients and be applied to the clinic practice to provide help to guide the treatment of patients. In our ABCC system score, the high-risk score had a worse prognosis than low-risk score in the stage IV patients, which revealed that the ABCC prognostic system had a widespread potential to applied into clinical than sole CCR index.

This study prioritized patients’ clinical manifestations and tumor progression, offering a clearer reflection of patient heterogeneity and individual differences compared to other studies. Clinical data are usually easy to obtain and more suitable for clinical application and decision support (36–38). However, this study has its own limitations. First, it is an observational retrospective study, which may be affected by selective bias. Predictive models should be validated in different cohorts to ensure robustness. Second, while this study explored the role of CCR in relation to nutritional status, the relationship between CCR and cachexia was not thoroughly investigated, particularly due to the absence of a definitive diagnosis of cachexia in advanced CRC patients, especially within the two years after surgery. Furthermore, sarcopenia-specific measures such as CT-defined muscle area or grip strength were not included, which limits the ability to fully assess muscle loss and its correlation with cachexia. However, we observed that the high CCR group showed elevated levels of weight, albumin, hemoglobin (HGB), and BMI, which are all correlated with nutritional status, suggesting that CCR levels may offer some indication of nutritional status in cancer patients. Third, most patients with distant metastases in this study were either resectable or had metastases occurring six months postoperatively, and patients with unresectable distant metastases were not included. While the general applicability of the biomarkers discussed here could be extended to earlier stages of CRC, further validation through prospective studies would be required to determine their effectiveness and utility in non-metastatic or early-stage CRC populations. Lastly, we plan to actively conduct multicenter research to further enrich the data with patients from different stages of disease and to validate the predictive consistency of the ABCC nutritional scoring system in an external cohort.

## 5 Conclusion

Our findings reveal that patients manifesting elevated CCR levels concurrently exhibited superior nutritional status and a more favorable prognosis in comparison to their counterparts with lower CCR levels. Additionally, we have introduced a comprehensive nutritional prognostic system score that integrates CCR levels, BMI, albumin, and CEA levels, dubbed the ABCC scores. This innovative system offers a more tailored approach for the prognostic assessment of individuals battling advanced metastatic CRC, thereby representing a significant leap forward in the personalized management of CRC.

## Data Availability

The raw data supporting the conclusions of this article will be made available by the authors, without undue reservation.
