# Supplementary material for: Assessing the prognostic value of serum creatinine to cystatin C ratio in stage III-IV colorectal cancer: development of a nutritional prognostic scoring system
Source: Front Oncol. 2025 Oct 21;15:1667472. doi: 10.3389/fonc.2025.1667472 (PMC12582935; doi:10.3389/fonc.2025.1667472)
Supplement: Supplementary file 1 [file Table1.docx]

Table S1: Different clinical parameters of high and low CCR stratified by gender

| **Characteristics** | **Female** | | | **Male** | | |
| --- | --- | --- | --- | --- | --- | --- |
|  | **high** | **low** | **P value** | **high** | **low** | **P value** |
|  | ***N=576*** | ***N=720*** |  | ***N=977*** | ***N=763*** |  |
| Tumor site: |  |  | 0.058 |  |  | 0.080 |
| Colon cancer | 251 (43.6%) | 353 (49.0%) |  | 420 (43.0%) | 361 (47.3%) |  |
| Rectal cancer | 325 (56.4%) | 367 (51.0%) |  | 557 (57.0%) | 402 (52.7%) |  |
| age | 57.0 [48.0;65.0] | 62.0 [54.0;70.0] | <0.001 | 58.0 [49.0;66.0] | 63.0 [55.5;72.0] | <0.001 |
| NRS2002: |  |  | 0.971 |  |  | 0.581 |
| 0 | 53 (9.20%) | 66 (9.17%) |  | 103 (10.5%) | 93 (12.2%) |  |
| 1 | 228 (39.6%) | 289 (40.1%) |  | 381 (39.0%) | 283 (37.1%) |  |
| 2 | 159 (27.6%) | 203 (28.2%) |  | 253 (25.9%) | 208 (27.3%) |  |
| 3 | 136 (23.6%) | 162 (22.5%) |  | 240 (24.6%) | 179 (23.5%) |  |
| Caprini: |  |  | 0.772 |  |  | 0.324 |
| 0-2 | 105 (18.2%) | 125 (17.4%) |  | 182 (18.6%) | 164 (21.5%) |  |
| 3-4 | 109 (18.9%) | 147 (20.4%) |  | 219 (22.4%) | 162 (21.2%) |  |
| >=5 | 362 (62.8%) | 448 (62.2%) |  | 576 (59.0%) | 437 (57.3%) |  |
| ECOG: |  |  | 0.272 |  |  | 1.000 |
| 0-1 | 32 (5.56%) | 52 (7.22%) |  | 60 (6.14%) | 46 (6.03%) |  |
| >=2 | 544 (94.4%) | 668 (92.8%) |  | 917 (93.9%) | 717 (94.0%) |  |
| albumin | 42.6 [37.0;45.7] | 41.1 [36.0;44.6] | <0.001 | 43.1 [39.0;46.1] | 41.0 [36.7;44.2] | <0.001 |
| immunoglobulin | 27.0 [24.0;30.0] | 27.2 [24.2;30.5] | 0.327 | 25.7 [23.1;29.0] | 26.6 [23.4;30.2] | <0.001 |
| AFP | 2.36 [1.70;3.42] | 2.48 [1.77;3.49] | 0.233 | 2.46 [1.77;3.37] | 2.61 [1.83;3.66] | 0.016 |
| CEA | 1.62 [1.12;2.59] | 1.69 [1.18;2.84] | 0.089 | 1.66 [1.18;2.47] | 1.82 [1.29;2.71] | <0.001 |
| CA199 | 15.5 [8.06;33.9] | 16.8 [9.05;41.0] | 0.060 | 14.3 [7.46;28.8] | 15.9 [8.15;32.8] | 0.077 |
| CA724 | 3.19 [1.54;7.28] | 3.48 [1.59;8.68] | 0.214 | 2.91 [1.54;8.68] | 3.54 [1.58;9.59] | 0.109 |
| CA125 | 12.3 [8.81;19.3] | 12.8 [8.95;20.7] | 0.237 | 10.8 [7.81;15.6] | 11.7 [8.26;18.6] | <0.001 |
| HGB | 118 [103;130] | 116 [101;129] | 0.108 | 139 [121;150] | 132 [113;146] | <0.001 |
| PLT | 221 [177;284] | 220 [169;272] | 0.106 | 206 [164;258] | 199 [154;256] | 0.027 |
| CCR | 1.23 [1.13;1.39] | 0.85 [0.71;0.95] | <0.001 | 1.27 [1.16;1.43] | 0.88 [0.78;0.98] | <0.001 |
| TNM: |  |  | 0.001 |  |  | <0.001 |
| III | 444 (77.1%) | 494 (68.6%) |  | 722 (73.9%) | 495 (64.9%) |  |
| IV | 132 (22.9%) | 226 (31.4%) |  | 255 (26.1%) | 268 (35.1%) |  |
| CCR grade: |  |  | <0.001 |  |  | 0.000 |
| Q1 | 0 (0.00%) | 406 (56.4%) |  | 0 (0.00%) | 361 (47.3%) |  |
| Q2 | 23 (3.99%) | 314 (43.6%) |  | 20 (2.05%) | 402 (52.7%) |  |
| Q3 | 306 (53.1%) | 0 (0.00%) |  | 446 (45.6%) | 0 (0.00%) |  |
| Q4 | 247 (42.9%) | 0 (0.00%) |  | 511 (52.3%) | 0 (0.00%) |  |
| dMMR: |  |  | 0.129 |  |  | 0.032 |
| dMMR | 40 (6.94%) | 68 (9.44%) |  | 51 (5.22%) | 60 (7.86%) |  |
| pMMR | 536 (93.1%) | 652 (90.6%) |  | 926 (94.8%) | 703 (92.1%) |  |

NRS2002, Nutrition Risk Screening 2002; Caprini, Caprini Risk Assessment Model; ECOG, Eastern Cooperative Oncology Group performance status; AFP, Alpha-Fetoprotein; CEA, Carcinoembryonic Antigen; CA199, Carbohydrate Antigen 19-9; CA724, Carbohydrate Antigen 72-4; CA125, Cancer Antigen 125; HGB, Hemoglobin; PLT, Platelet; CCR, creatinine to cystatin C ratio; dMMR, Deficient Mismatch Repair; pMMR, Proficient Mismatch Repair.

Table S2: Different clinical parameters of high and low CCR stratified by TNM stage

| **Characteristics** | **III stage** | | | **IV stage** | | |
| --- | --- | --- | --- | --- | --- | --- |
|  | **high** | **low** | **P value** | **high** | **low** | **P value** |
|  | ***N=1166*** | ***N=989*** |  | ***N=387*** | ***N=494*** |  |
| Surgical Technique |  |  | 0.951 |  |  | 0.006 |
| Laparoscope | 739 (63.4%) | 629 (63.6%) |  | 135 (34.9%) | 129 (26.1%) |  |
| Open surgery | 427 (36.6%) | 360 (36.4%) |  | 252 (65.1%) | 365 (73.9%) |  |
| Tumor site |  |  | 0.067 |  |  | 0.445 |
| colon cancer | 457 (39.2%) | 427 (43.2%) |  | 214 (55.3%) | 287 (58.1%) |  |
| rectal cancer | 709 (60.8%) | 562 (56.8%) |  | 173 (44.7%) | 207 (41.9%) |  |
| gender: |  |  | <0.001 |  |  | 0.001 |
| female | 444 (38.1%) | 494 (49.9%) |  | 132 (34.1%) | 226 (45.7%) |  |
| male | 722 (61.9%) | 495 (50.1%) |  | 255 (65.9%) | 268 (54.3%) |  |
| age | 58.0 [49.0;65.0] | 63.0 [55.0;72.0] | <0.001 | 56.0 [49.0;65.0] | 61.0 [54.0;71.0] | <0.001 |
| NRS2002: |  |  | 0.853 |  |  | 0.635 |
| 0 | 122 (10.5%) | 112 (11.3%) |  | 34 (8.79%) | 47 (9.51%) |  |
| 1 | 465 (39.9%) | 391 (39.5%) |  | 144 (37.2%) | 181 (36.6%) |  |
| 2 | 318 (27.3%) | 276 (27.9%) |  | 94 (24.3%) | 135 (27.3%) |  |
| 3 | 261 (22.4%) | 210 (21.2%) |  | 115 (29.7%) | 131 (26.5%) |  |
| Caprini: |  |  | 0.578 |  |  | 0.401 |
| 0-2 | 214 (18.4%) | 192 (19.4%) |  | 73 (18.9%) | 97 (19.6%) |  |
| 3-4 | 240 (20.6%) | 215 (21.7%) |  | 88 (22.7%) | 94 (19.0%) |  |
| >=5 | 712 (61.1%) | 582 (58.8%) |  | 226 (58.4%) | 303 (61.3%) |  |
| ECOG: |  |  | 0.480 |  |  | 1.000 |
| 0-1 | 67 (5.75%) | 65 (6.57%) |  | 25 (6.46%) | 33 (6.68%) |  |
| >=2 | 1099 (94.3%) | 924 (93.4%) |  | 362 (93.5%) | 461 (93.3%) |  |
| albumin | 43.2 [38.5;46.1] | 41.5 [37.0;44.8] | <0.001 | 41.8 [37.5;45.1] | 40.2 [35.6;43.0] | <0.001 |
| immunoglobulin | 25.9 [23.4;29.3] | 26.7 [23.6;30.0] | 0.006 | 26.3 [23.3;29.7] | 27.3 [24.0;31.1] | 0.004 |
| AFP | 2.41 [1.72;3.40] | 2.61 [1.86;3.58] | 0.001 | 2.47 [1.79;3.38] | 2.46 [1.66;3.59] | 0.569 |
| CEA | 1.56 [1.11;2.20] | 1.60 [1.17;2.38] | 0.025 | 2.11 [1.35;3.68] | 2.29 [1.39;3.96] | 0.248 |
| CA199 | 13.7 [7.49;25.7] | 14.2 [8.10;27.3] | 0.194 | 20.0 [8.41;75.3] | 22.7 [9.22;113] | 0.110 |
| CA724 | 3.13 [1.52;8.68] | 3.42 [1.58;8.68] | 0.248 | 2.91 [1.55;7.01] | 3.59 [1.59;10.5] | 0.060 |
| CA125 | 10.8 [7.83;15.5] | 11.4 [8.00;17.3] | 0.012 | 13.0 [9.18;22.2] | 14.9 [9.77;30.1] | 0.010 |
| HGB | 133 [113;146] | 126 [107;140] | <0.001 | 124 [106;139] | 120 [102;136] | 0.029 |
| PLT | 212 [171;265] | 210 [162;266] | 0.236 | 211 [159;273] | 204 [151;265] | 0.099 |
| CCR | 1.26 [1.15;1.42] | 0.87 [0.76;0.98] | 0.000 | 1.23 [1.13;1.39] | 0.85 [0.73;0.96] | <0.001 |
| CCR grade: |  |  | 0.000 |  |  | <0.001 |
| Q1 | 0 (0.00%) | 488 (49.3%) |  | 0 (0.00%) | 279 (56.5%) |  |
| Q2 | 30 (2.57%) | 501 (50.7%) |  | 13 (3.36%) | 215 (43.5%) |  |
| Q3 | 549 (47.1%) | 0 (0.00%) |  | 203 (52.5%) | 0 (0.00%) |  |
| Q4 | 587 (50.3%) | 0 (0.00%) |  | 171 (44.2%) | 0 (0.00%) |  |
| dMMR: |  |  | 0.025 |  |  | 0.142 |
| dMMR | 65 (5.57%) | 80 (8.09%) |  | 26 (6.72%) | 48 (9.72%) |  |
| pMMR | 1101 (94.4%) | 909 (91.9%) |  | 361 (93.3%) | 446 (90.3%) |  |

NRS2002, Nutrition Risk Screening 2002; Caprini, Caprini Risk Assessment Model; ECOG, Eastern Cooperative Oncology Group performance status; AFP, Alpha-Fetoprotein; CEA, Carcinoembryonic Antigen; CA199, Carbohydrate Antigen 19-9; CA724, Carbohydrate Antigen 72-4; CA125, Cancer Antigen 125; HGB, Hemoglobin; PLT, Platelet; CCR, creatinine to cystatin C ratio; dMMR, Deficient Mismatch Repair; pMMR, Proficient Mismatch Repair.
